# Establishment and characterization of a new human colon cancer cell line, PUMC-CRC1

**DOI:** 10.1038/s41598-021-92491-7

**Published:** 2021-06-23

**Authors:** Xiaocui Bian, Fang Cao, Xiaowan Wang, Yuhong Hou, Haitao Zhao, Yuqin Liu

**Affiliations:** 1 Department of Pathology, Cell Resource Center, Institute of Basic Medical Sciences, Chinese Academy of Medical Sciences (CAMS) & School of Basic Medicine, Peking Union Medical College (PUMC), Beijing, 100005 People’s Republic of China; 2 Key Laboratory of Carcinogenesis and Translational Research (Ministry of Education), Department of Pathology, Peking University Cancer Hospital & Institute, Beijing, 100142 People’s Republic of China; 3 Department of Liver Surgery, Peking Union Medical College Hospital, Chinese Academy of Medical Sciences and Peking Union Medical College (CAMS & PUMC), Beijing, 100730 People’s Republic of China

**Keywords:** Cancer models, Cancer models, Colon cancer

## Abstract

Colorectal cancer (CRC) is one of the most common and fatal gastrointestinal cancers worldwide. Considering their diversity, the establishment of new continuous CRC cell lines with clear genetic backgrounds will provide useful tools for exploring molecular mechanisms, screening and evaluating antitumor drugs in CRC studies. Our de novo CRC cell line, PUMC-CRC1 (Peking Union Medical College Colorectal Cancer 1) was derived from a 47-year-old Chinese female patient diagnosed with moderately to poorly differentiated colon adenocarcinoma. Multiple experiments were used for full characterization. The new cell line was epithelial-like and was passaged for more than 40 times, with a population doubling time of 44 h in vitro, detected by cell counts. The cells exhibited complicated chromosomal abnormalities. The tumor formation rate in SCID mice was 100%. The xenograft tumor was adenocarcinoma with poor to moderate differentiation by Haematoxylin and Eosin staining (H&E) sections. Immunohistochemistry (IHC) analysis and next-generation sequencing (NGS) revealed microsatellite stable (MSS), APC (p.T1493fs) inactivation, KRAS (p.G12V) activation, and SMAD4 (p.V506A) mutation. Quality control of the cell line proved mycoplasma negative and identical STR profile with that of the original tissue, and no interspecific or intraspecific cross contamination was detected. In conclusion, PUMC-CRC1 was a newly established and well characterized human colon cancer cell line, which might be a good model for both in vitro and in vivo studies of the mechanism of colon cancer progression and the treatment strategies for MSS CRC.

## Introduction

CRC is one of the most common gastrointestinal cancers worldwide, with the third highest incidence rate and third highest mortality rate^1–5^. It is a heterogeneous disease of the intestinal epithelium that is characterized by the accumulation of mutations and a dysregulated immune response^6–8^. There are three major molecular carcinogenesis pathways of CRC: (1) the traditional adenoma–carcinoma pathway, also referred to as chromosomal instability (CIN) (70–90% of CRC), characterized by abnormal karyotypes, aneuploidy, and the loss of heterozygosity, causing MSS tumors, which are initiated by inactivation of the Adenomatous Polyposis Coli (*APC*) tumor suppressor gene (> 80% of cases) and followed by activating mutations of *KRAS* (approximately 50% of cases) and subsequent malignant transformation driven by additional mutations in the TGF-β, PIK3CA, and TP53 pathways; (2) the serrated neoplasia pathway (10–20% of CRC), characterized by the CpG island methylation phenotype (CIMP), leading to microsatellite stable and instable cancers; and (3) microsatellite instability (MSI) (2–7% of CRC), initiated by the germline mutation of mismatch repair genes such as *MSH2*, *MSH6*, *MLH1*, *MLH6*, *PMS1* and *PMS2*, that is also seen in Lynch syndrome and characterized by the silencing of DNA repair mechanisms^4,9–11^. On the basis of gene expression, different types of CRC have different treatment strategies and prognoses.

A well established CRC cell line with a clear genetic background, as a pure, renewable population, can be used as a practical model for biological and molecular research, anticancer drug screening, and diagnosis and biomarker identification^9,12–14^. However, the process of cell line establishment from fresh tumor tissues is time-consuming and difficult due to contamination, out-growth of cancer-associated fibroblasts and cellular senescence. Therefore, the success rate of cell line establishment from fresh tumor tissues is around 10% according to our experience.

In present study, we established a new cell line, designated PUMC-CRC1, from the fresh tumor tissue of a 47-year-old Chinese female patient with Duke's C1 and moderately to poorly differentiated adenocarcinoma in the cecum, who underwent right hemicolectomy and didn’t receive any chemotherapy or radiotherapy before and after surgery. Quality control is essential for cell culture^15,16^. During the process of the establishment of the PUMC-CRC1 cell line, samples were repeatedly collected for cell species identification, STR analysis and Mycoplasma detection, to ensure that there was no contamination with other cells or foreign microorganisms. The cell morphology, growth characteristics, cytogenetic features, cancer-related gene mutations, in vivo tumorigenicity and IHC markers of the xenografts were characterized. This newly established cell line should serve as a useful model for in vitro and in vivo studies of the carcinogenic mechanisms and screening for new therapeutic drugs against colorectal cancer.

## Results

### Morphology and growth characteristics of the PUMC-CRC1 cell line

**Figure 1:**
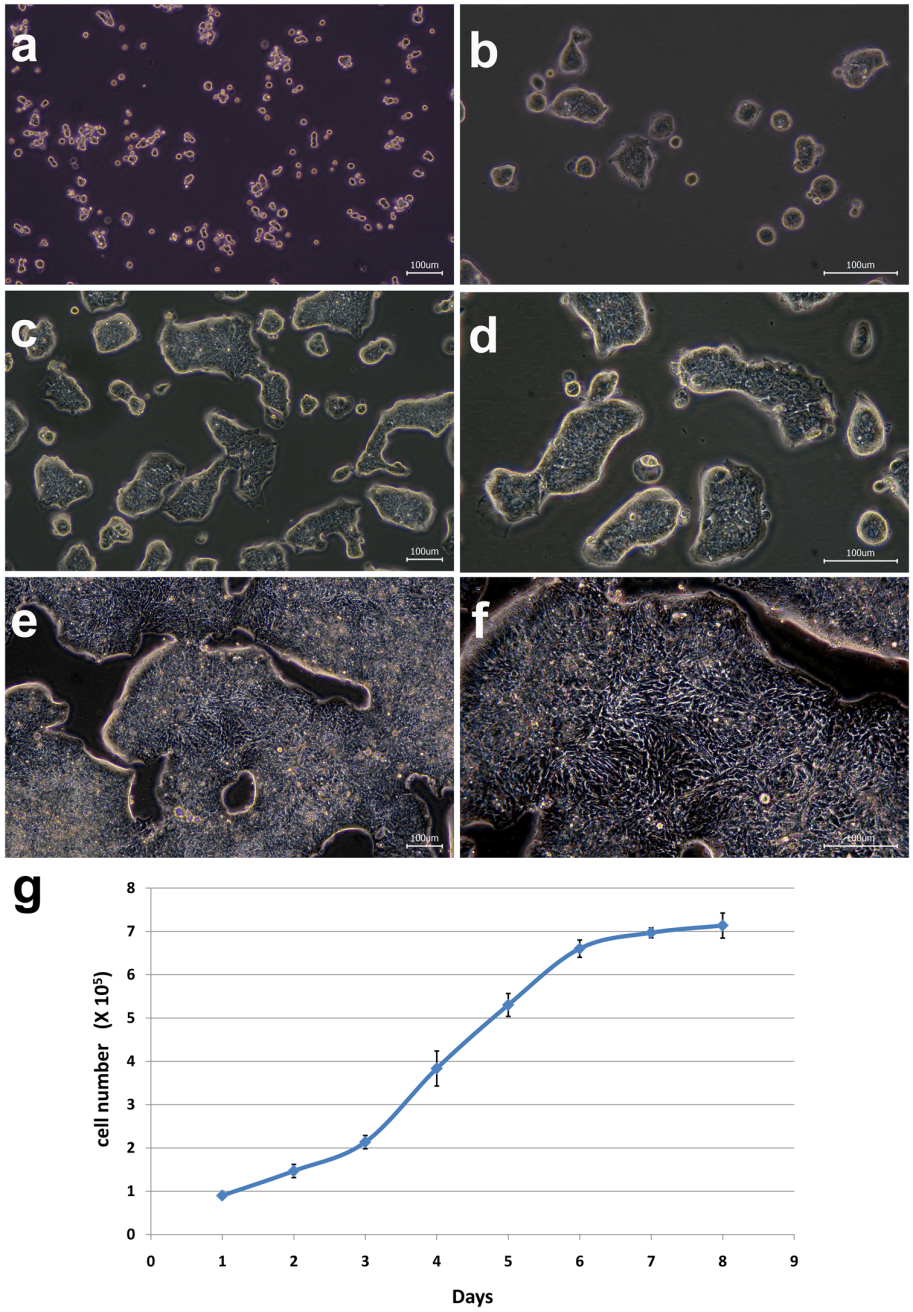
Growth characteristics of the PUMC-CRC1 cells. (**a**, **b**) Morphological images of PUMC-CRC1 at low density under low and high magnification respectively. (**c**, **d**) Morphological images of PUMC-CRC1 at medium density under low and high magnification respectively. (**e**, **f**) Morphological images of PUMC-CRC1 at high density under low and high magnification respectively. (**c**) Growth curve of PUMC-CRC1 cells at P22 using cell counts.

In order to remove cancer-associated fibroblasts, G418 (100 ug/ml) was used for 2 weeks in primary culture. All fibroblasts and most tumor cells were killed, and only one visible clone survived 2 months later, which suggested that the concentration and exposure time of G418 also affected the survival of tumor cells. It took nearly 7 months for this clone to grow and be passaged. To date, the PUMC-CRC1 cell line has been passaged in vitro for more than 40 passages. Under an inverted phase microscope the cells adhered firmly to the culture flask, grew in island and were epithelial (Fig. 1a–f). The cells grew relatively slowly in vitro, with a population doubling time of 44 h (Fig. 1g). After recovery from liquid nitrogen, cryopreserved cells could be propagated in culture without noticeable changes in growth and morphology. The established cell line did not exhibit any signs of senescence and it was therefore considered immortalized. No signs of fungal or bacterial contamination were observed. Both culture- and PCR-based methods indicated that there was no Mycoplasma in the culture of the PUMC-CRC1 cells.

### Identity authentication

PUMC-CRC1 was confirmed as human origin by a PCR-based method (Supplementary Figs. S1 and S2). Then, DNA samples from different passages (P3, P21, P26 and the original tumor tissues) were subjected to STR profiling. A total of 18 STR loci and the amelogenin sex-determining marker were co-amplified in each sample.

The data were analyzed and allele(s) of each locus were determined (Table 1). The STR profiles of PUMC-CRC1 cells were basically the same as those of the original tumor and unique when compared with data published on the DSMZ (German Collection of Microorganisms and Cell Cultures) and BMCR (Biomedical Cell Resource Center) online STR databases, suggesting that the PUMC-CRC cells were authentic. However, additional genetic aberrations were observed at loci D8S1179 and TH01 during the passage.

**Table 1:** STR profiles of the original tumor tissues and PUMC-CRC1 cells at different passages.

Locus	Original tumor tissues	P3	P21	P26
Amelogenin	X,X	X,X	X,X	X,X
CSF1PO	12,12	12,12	12,12	12,12
D12S391	17,18	17,18	17,18	17,18
D13S317	8,10	8,10	8,10	8,10
D16S539	10,10	10,10	10,10	10,10
D18S51	16,16	16,16	16,16	16,16
D19S433	13,13.2	13,13.2	13,13.2	13,13.2
D21S11	30,32	30,32	30,32	30,32
D2S1338	18,24	18,24	18,24	18,24
D3S1358	15,16	15,16	15,16	15,16
D5S818	11,11	11,11	11,11	11,11
D6S1043	11,14	11,14	11,14	11,14
D7S820	7,11	7,11	7,11	7,11
D8S1179	13,13	13,15	13,15	13,15
FGA	20,20	20,20	20,20	20,20
Penta E	17,17	17,17	17,17	17,17
TH01	7,7	7,9	7,9	7,9
TPOX	8,9	8,9	8,9	8,9
vWA	16,16	16,16	16,16	16,16

### Chromosome analysis

**Figure 2:**
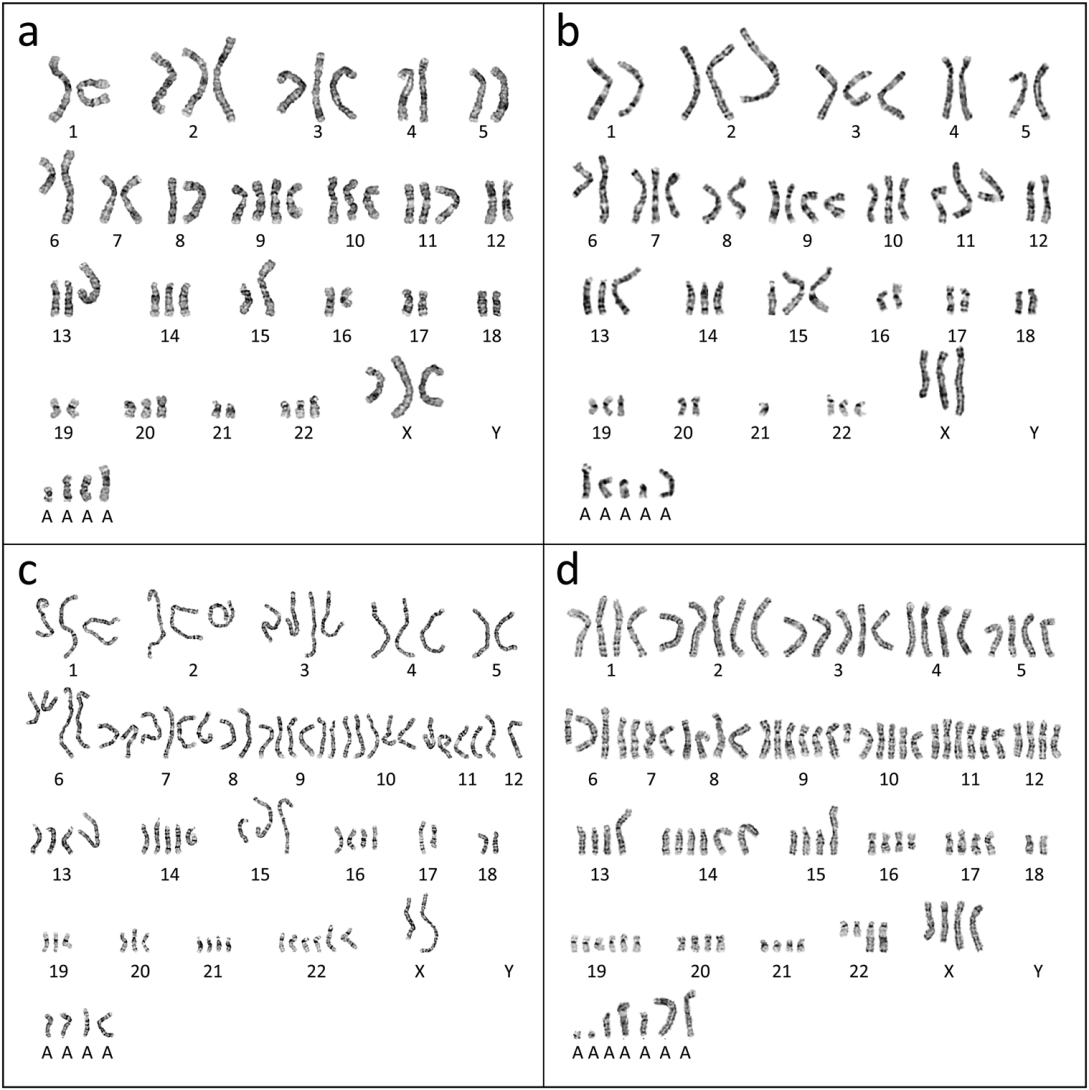
Chromosome analysis of PUMC-CRC1 cells at passage 34. (**a**, **b**) Representative karyotypes of hyperdiploid cells, accounting for 65% of PUMC-CRC1 cells at P34, with chromosomal numbers ranging from 60 to 68. (**c**) Representative karyotypes of hypertriploid cells, accounting for 15% of PUMC-CRC1 cells at P34, with chromosomal numbers ranging from 89 to 93. (**d**) Representative karyotypes of hypertetraploid cells, accounting for 20% of PUMC-CRC1 cells at P34, with chromosomal numbers ranging from 101 to 110.

The complicated karyotype and abnormal chromosome number of the PUMC-CRC1 cells were revealed by chromosome analysis. Most cells (65%) examined at passage 34 were hyperdiploid (Fig. 2a,b), with chromosomal numbers ranging from 60 to 68. In addition, 15% and 20% of cells were hypertriploid (Fig. 2c) and hypertetraploid (Fig. 2d), with chromosomal numbers ranging from 89 to 93 and 101 to 110, respectively. Each subgroup carried the same structural chromosomal abnormalities, but the number of chromosomes (normal and abnormal) was different. According to the composite karyotype in the ISCN (International System for Human Cytogenomic Nomenclature), the representative karyotype of the hyperdiploid cells was named 60 ~ 68 < 3n > , X, − X, add(X)(q22)*2, − 1,add(1)(p36), + 2, der(2)t(1;2)(q21;p25), − 4, − 5, − 6, add(6)(q12), add(6)(q12), -8,add(8)(p11.2), + 9, + 9, − 11, add(11)(p11.2), − 12,add(13)(p11.2), idic(15)(p12)*2, − 16, del(16)(q22), − 17, add(17)(q25), − 18, − 18, − 19, add(19)(p13), − 20, − 21, add(21)(q21), + 22, − 22, add(22)(q11.2)*2, + 2 ~ 8mar.

### Tumorigenicity in SCID mice

**Figure 3:**
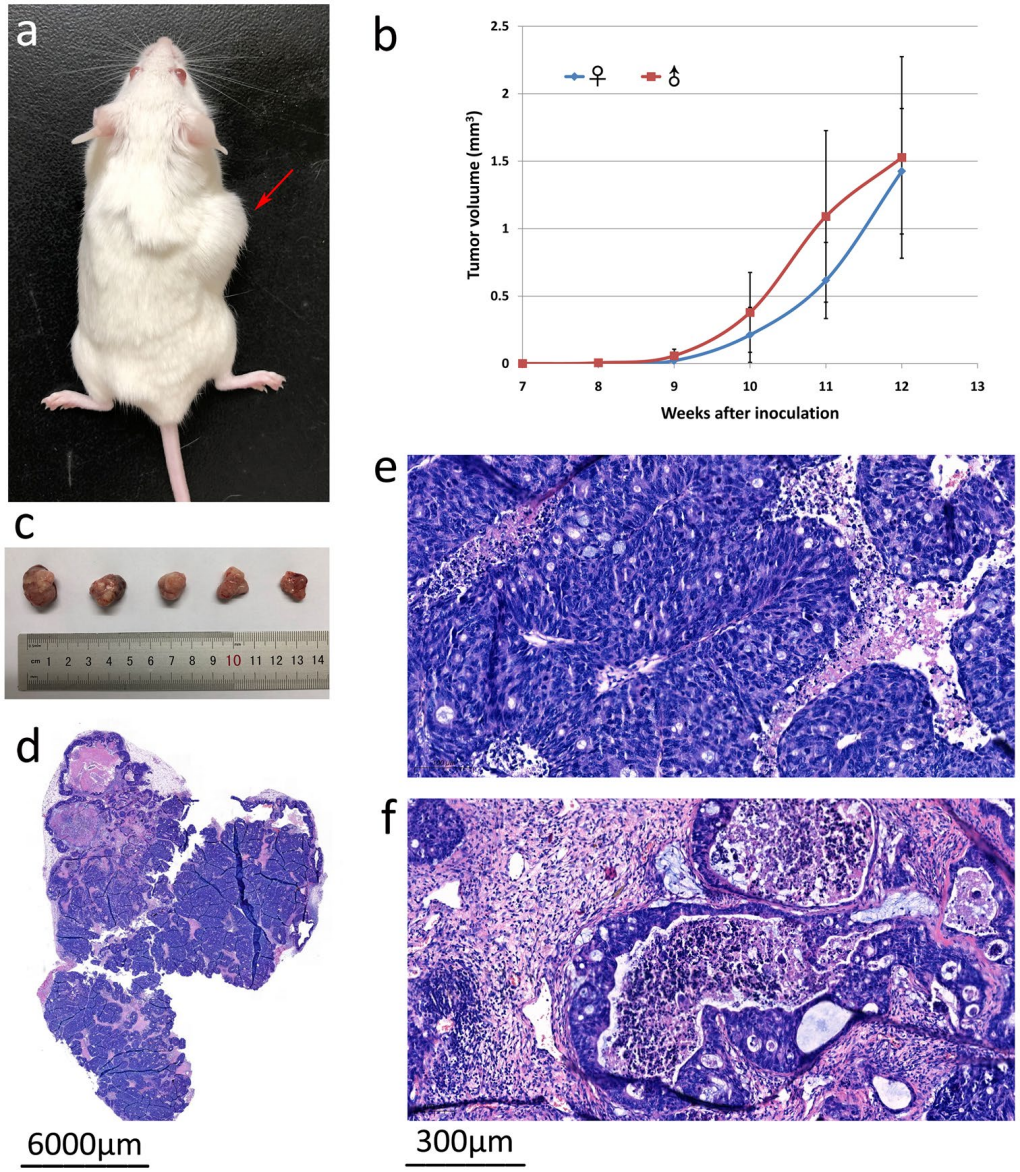
In vivo size assessment and histological analysis of the xenograft tumors in SCID mice. (**a**) Subcutaneous tumor mass (arrow indicated) in SCID mice which inoculated with 6 × 10^6^ PUMC-CRC1 cells at 3 months post-injection. (**b**) Growth curve of the xenograft tumors in female and male SCID mice (n = 5, age 5–6 weeks), which started to increase in size at around 9 weeks after inoculation. (**c**) Xenografts derived from PUMC-CRC1 cells. (**d–f**) H&E staining of the paraffin-embedded tissue section confirmed that the xenograft tumor was moderately to poorly differentiated colorectal adenocarcinoma: (**d**) whole slide image of the xenograft tumor under low magnification; (**e**) irregular glandular cavity formation and cribriform structure; (**f**) cribriform structure and “dirty necrosis” within the glandular lumina.

About 9 weeks after the inoculation of PUMC-CRC1 cells in female SCID mice, subcutaneous tumor masses were palpable at the site of inoculation, and the diameter of the tumor was close to 1.5 cm at 3 months post-injection (Fig. 3a–c). The tumor formation rate was 100%. The same results were obtained in male SCID mice. Compared by *t* test, there was no significant difference in tumor size measured at the same time point after inoculation between males and females (*p*-value was 0.172, 0.190, 0.329, 0.165 and 0.807 at 8, 9, 10, 11, 12 weeks post-injection, respectively).

### Pathology analysis

H&E stained sections showed that the subcutaneous xenograft tumors were moderately to poorly differentiated colorectal adenocarcinomas with irregular glandular cavity formation, cribriform structure and “dirty necrosis” within the glandular lumina, which is often considered characteristic of CRC (Fig. 3d–f). Examined by the same pathologist, the differentiation of the xenograft was consistent with that of the original tumor samples.

**Figure 4:**
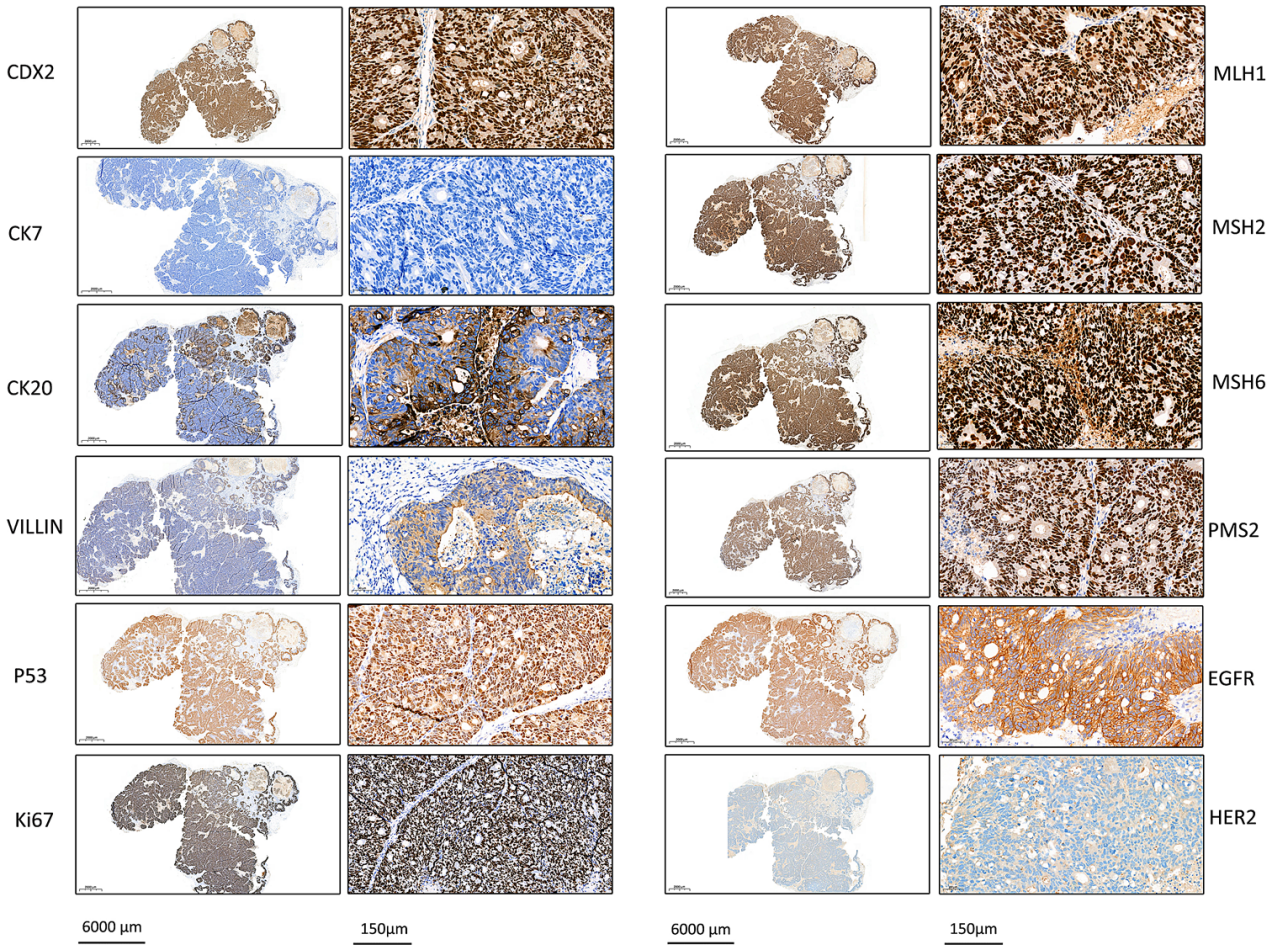
IHC analysis of PUMC-CRC1 xenografts. Four characteristic markers of CRC (CDX2, CK7, CK20 and VILLIN) and 8 biomarkers related to prognosis and treatment (P53, Ki67, EGFR, HER2, MLH1, PMS2, MSH2 and MSH6) were detected by IHC. Whole slide IHC image under low magnification and representative IHC image under high magnification of each marker were showed in left and right respectively.

Four characteristic markers of CRC were detected by IHC in the xenografts. The results were CDX2(+), CK7(−), CK20(partial+) and VILLIN(partial+), which supported the phenotype of primary colorectal adenocarcinoma. Meanwhile, 8 biomarkers related to prognosis and treatment were tested. The results showed that P53 was diffusely positive, and the proliferation index marker Ki67 was 80% in the xenografts, suggesting poor prognosis. The expression of mismatch repair proteins, including MLH1, PMS2, MSH2 and MSH6, was positive suggesting that there was no defect in the tumor. In addition, EGFR and HER2/Neu staining were both moderately positive and consistent with the results of gene sequencing, which indicated that there was no EGFR or HER2 amplification in the tumor (Fig. 4).

### Gene mutations in PUMC-CRC1 cells

Screening by NGS, sixteen cancer related gene mutations were detected and listed in Table 2. Among them, 5 mutated genes, *APC*, *KRAS*, *SMAD4*, *ERBB4* and *PTK2,* were detected in PUMC-CRC1 cells, not in normal tissues of the same patient, and considered as somatic mutations. A frameshift mutation in exon 16 of *APC* gene (the mutation abundance was 89.33%) was detected in PUMC-CRC1 cells, which resulted in the disruption of reading frame and predicted the inactivation of APC function. A missense mutation in exon 2 of *KRAS* gene, with the mutation abundance 47.44% in PUMC-CRC cells, resulted in KRAS (p.G12V) activation. The abundances of SMAD4 (p.V506A), ERBB4 (p.D564Y), ERBB4 (p.V857L) and PTK2 (p.L424P) mutations were 94.11%, 28.27%, 28.36% and 30.79% respectively, and change of protein functions caused by these mutations was unknown. Eleven mutated genes were detected in both PUMC-CRC1 cells and the normal tissues. Among these genes, homozygous mutations were found in *AXL*, *FAM46C*, and *MST1R*, and other mutations were heterozygous. No mutations were found in some important genes for colorectal cancer development, such as *BRAF*, *PIK3CA*, *PTEN*, *TP53, EGFR* and mismatch repair (MMR) genes (MLH1, PMS2, MSH2 and MSH6) (Table 3). The results of gene mutations suggested that PUMC-CRC1 was derived from a sporadic MSS CRC, with consistent IHC results. Comparing the mutation status of cancer critical genes in PUMC-CRC1 with that in 19 commonly-used CRC cell lines, it was proved that PUMC-CRC1 with APC (p.T1493fs), KRAS (p.G12V) and SMAD4 (p.V506A) mutations was a unique MSS CRC cell line (Table 3).

**Table 2:** Sixteen cancer related gene mutations in PUMC-CRC1 cells and the normal tissues of the same patient.

Gene	Position	CDS mutation	AA mutation	Type	PUMC-CRC1	normal tissues
*APC*	5q22.2, exon16	c.4476_4479del CACGinsACA	p.T1493fs	Deletion—Frameshift	Yes	No
*KRAS*	12p12.1, exon2	c.35G>T	p.G12V	Substitution—Missense	Yes	No
*SMAD4*	18q21.2, exon12	c.1517T>C	p.V506A	Substitution—Missense	Yes	No
*ERBB4*	2q34,exon14	c.1690G>T	p.D564Y	Substitution—Missense	Yes	No
	2q34,exon21	c.2569G>T	p.V857L	Substitution—Missense	Yes	No
*PTK2*	8q24.3, exon16	c.1271T>C	p.L424P	Substitution—Missense	Yes	No
*AR*	chrX:66765516	c.528C>A	p.S176R	Substitution—Missense	Yes	Yes
*ARID1A*	chr1:27057887	c.1595C>T	p.A532V	Substitution—Missense	Yes	Yes
*AXL*	chr19:41743861	c.796A>G	p.N266D	Substitution—Missense	Yes	Yes
*FAM46C*	chr1:118165691	c.201C>G	p.H67Q	Substitution—Missense	Yes	Yes
*MST1R*	chr3:49928691	c.3583A>G	p.S1195G	Substitution—Missense	Yes	Yes
*PARP1*	chr1:226567698	c.1468G>A	p.V490I	Substitution—Missense	Yes	Yes
*RAD50*	chr5:131915043	c.400G>A	p.A134T	Substitution—Missense	Yes	Yes
*ROS1*	chr6:117622231	c.6639C>A	p.D2213E	Substitution—Missense	Yes	Yes
*SETD2*	chr3:47163843	c.2283G>A	p.M761I	Substitution—Missense	Yes	Yes
*SH2B3*	chr12:111886081	c.1703T>C	p.I568T	Substitution—Missense	Yes	Yes
*TCF7L2*	chr10:114925495	c.1573C>G	p.P525A	Substitution—Missense	Yes	Yes

Four genes (*APC*, *KRAS*, *SMAD4*, *ERBB4* and *PTK2*) were mutated in PUMC-CRC1 cells, not in normal tissues of the same patient, and 11 gene mutations were detected in both PUMC-CRC1 cells and the normal tissues.

**Table 3:** Comparison of MSI status and mutation status of cancer critical genes in PUMC-CRC1 and 19 commonly-used CRC cell lines.

Cell line	MSI status	APC	K-RAS	BRAF	PIK3CA	PTEN	TP53	SMAD4	MLH1	PMS2	MSH2	MSH6
PUMC-CRC1	MSS	p.T1493fs	p.G12V	wt	wt	wt	wt	p.V506A	wt	wt	wt	wt
Caco-2	MSS	p.Q1367*	wt	wt	wt	wt	p.C135F; p.E204*	p.D351H	wt	wt	wt	wt
Colo201	MSS	p.E1554fs	wt	p.V600E	wt	wt	wt	wt	wt	p.E504Q	wt	wt
Colo205	MSS	p.E1554fs	wt	p.V600E	wt	wt	wt	wt	wt	p.E504Q	wt	wt
Colo320	MSS	p.S811*	wt	wt	wt	wt	p.R248W	wt	wt	wt	wt	wt
Colo678	MSS	p.E1554fs	p.G12D	wt	wt	wt	wt	wt	wt	wt	wt	wt
DIFI	MSS	p.E1151*; p.E443fs	wt	wt	wt	wt	p.K132R	wt	wt	wt	wt	wt
HT-29	MSS	p.E1554fs	wt	p.V600E	p.P449T	wt	p.V600E	p.Q311*	wt	wt	wt	wt
NCI-H508	MSS	wt	wt	p.G596R	p.E545K	wt	p.R273H	wt	wt	wt	wt	wt
SW1116	MSS	p.QTMP1429fs; p.Q264*	p.G12A	wt	wt	wt	p.A159D	wt	wt	p.D414fs	wt	wt
SW480	MSS	p.Q1338*	p.G12V	wt	wt	wt	p.R273H; p.P309S	wt	wt	wt	wt	wt
SW620	MSS	p.Q1338*	p.G12V	wt	wt	wt	p.R273H; p.P309S	wt	wt	wt	wt	wt
T84	MSS	p.L1489fs	p.G13D	wt	p.E542K	wt	p.?	p.K340N	wt	wt	wt	wt
CW-2	MSI-H	p.K1462fs; p.A528V; p.E2737G; p.I2756V; p.G470R; p.R302*	p.P140H	wt	p.P283S	p.P38H	p.P47P	wt	p.V152M; p.Y130fs	wt	wt	wt
HCT116	MSI-H	wt	p.G13D	wt	p.H1047R	wt	wt	wt	p.S252*	wt	wt	p.T1085fs
HCT-15	MSI-H	p.R727M; p.K993N; p.K1561N; p.I1779M; p.R2166*; p.E2550Q	p.G13D	wt	p.E545K; p.D549N	wt	p.S241F	wt	p.A120S; p.L348L	p.D543D	wt	p.T1189I; p.G289fs; p.DR1171fs; p.D1171fs; p.R1172del
HRT-18	MSI	p.R727M; p.K993N; p.R2166*; p.G1416fs	p.G13D; p.P110H	WT	p.E545K; p.D549N	p.Q87P; p.P96S	WT	p.D165E	p.A120S	wt	p.A600V	p.G289fs
LOVO	MSI-H	p.R1114*; p.R2816Q p.T1430fs	p.G13D	wt	wt	wt	wt	wt	wt	wt	wt	wt
LS180	MSI-H	p.R1788C; p.K2193K	p.G12D	p.D211G	p.H1047R	p.I67K	wt	wt	p.T117M	wt	wt	p.T1085fs
RKO	MSI-H	wt	wt	p.V600E	p.H1047R	wt	wt	wt	p.L323M	wt	wt	p.Y1066fs

Gene mutations of 19 commonly-used CRC cell lines were obtained at the Cancer Cell Lines Encyclopedia (CCLE, https://portals.broadinstitute.org/ccle), the Catalogue of Somatic Mutations in Cancer (COSMIC, https://cancer.sanger.ac.uk/cell_lines) and the Cellosaurus (https://web.expasy.org/cellosaurus/). Mutations are annotated at the protein level as described by Dunnen et al. (Hum Mutat. 2016;37(6):564–569. https://doi.org/10.1002/humu.22981), and one-letter amino acid code was used. *MSI-H* MSI-high, *wt* wild type, *fs* frame shift, *p.?* predictions, *del* deletion; *Stop codon.

## Discussion

According to cancer statistics from 2020, CRC is one of the most common malignancies and leading causes of cancer-related death worldwide^1,2^. Despite recent progress, to date, effective therapy and molecular mechanisms underlying carcinogenesis, metastasis and recurrence remain to be thoroughly studied and elucidated^3,4,17^.

Biomedical research on CRC relies largely on cellular and animal models. Cells established from surgical specimens may provide an invaluable alternative and renewable source of material^18–20^. As reported in the Cellosaurus (https://web.expasy.org/cellosaurus/), excluding derivative cell lines, there were 160 and 36 cancer cell lines derived from colon and rectum cancer samples respectively. Among them, a dozen cell lines were widely used for CRC research, HCT116, HCT-8, SW480, SW620, being the most frequently employed^21–24^. However, these cell lines show versatile morphological features and biochemical markers and represent only some of the biological types of CRC. Therefore, more cell models derived from human primary tumors with clear backgrounds are required for the study of CRC biology. Like other established cell lines, the novel PUMC1-CRC cells have some particular advantages, including: high phenotypic heterogeneity, decreased genetic shifts, ideal complements for tissue banks, new tools for the evaluation of anticancer agents, and endless sources for genetic or proteomic analysis^25–27^.

Successful establishment of new cell lines means hard work and good luck. As reported, the success rate of human colon cancer cell line from fresh tumors was 9.7%^28^. One of the main reasons for failure was the overgrowth of cancer-associated fibroblasts. Fibroblasts can be eliminated by differential adhesion, differential detachment and “Geneticin” treatment^29–31^. The mechanism of selective elimination depends on differences in the chemosensitivity of fibroblasts and cancer cells. Another reason for failure to establish a stable cell line was cellular senescence^32^. Although malignant cells are believed to be constitutively immortal as a consequence of neoplastic transformation, many in vitro cultures display a short life span as they undergo a limited number of cell passages before entering a state of irreversible growth arrest^33^. Telomorase reverse transcriptase (TERT), SV40 T-antigen and HPV E6/E7 are often used to immortalize cells^32–37^. These tools can help us obtain unlimited experimental materials, while at the same time provide some artificial background^33,34^. As reported, intrinsic gene changes determine the successful establishment of stable cancer cell lines from tumor tissue^38^. In this study, we obtained a sample with enough mutations to permit long term culture and the establishment of a continuous cell line without the transfer of exogenous genes, which will be more suitable for studying CRC. The PUMC-CRC1 cells displayed chromosomal instability providing rapid adaptation, which may be one factor leading to successful culture. Chromosomes of tumor cells in the early stage are mostly diploid, and chromosome instability increases with tumor progression and is often accompanied by changes in chromosome number and the generation of hybrid DNA^39,40^. Chromosomal deletion, cleavage and rearrangement lead to the activation of oncogenes or the inactivation of tumor suppressor genes, which promotes tumorigenesis and development^41,42^.

PUMC-CRC1 was a unique MSS CRC cell model. As a cell line derived from a cancer specimen, PUMC-CRC1 exhibits malignant behaviours both in vitro and in vivo, such as chromosomal abnormalities, unlimited proliferation, the loss of density dependence and tumor formation in SCID mice. Moreover, H&E and anti-CDX2, CK7, CK20 and VILLIN IHC staining of xenografts also confirmed its colorectal adenocarcinoma characteristics. A clear genetic background is important for cell line models in cancer research^21^, especially in the study of precision medicine and targeted drugs. Detected by NGS and IHC, somatic mutations of *APC*, *KRAS* and *SMAD4* and wild type of MMR genes combined with chromosomal instability confirmed that the PUMC-CRC1 was derived from a sporadic MSS CRC, which developed by the sequential accumulation of genetic mutations and chromosomal instability. Comparing the mutation status of cancer critical genes in PUMC-CRC1 with that in 19 commonly-used CRC cell lines, PUMC-CRC1 was a unique cell line with APC (p.T1493fs), KRAS (p.G12V) and SMAD4 (p.V506A) mutations, which enriches the diversity of CRC cell lines. Somatic *APC* mutations are found in more than 80% of sporadic colorectal tumors. Mutation and inactivation of *APC* is regarded as essential to the initiation of colon cancer through loss of APC tumor suppressive functions, such as regulating the canonical Wnt signaling pathway to control cell proliferation and differentiation, cell adhesion, and cell migration. As reported, the inactivated APC can lead to dysfunction in spindle formation and mitotic progression due to the lack of binding domains for microtubules, contributing to CIN and CRC progression^43^. Forty to forty-five percent of CRC are characterized by mutations in the KRAS proto-oncogene, most frequently affecting codons 12 (G12D, 13%; G12V, 9%) and 13 (G13D, 8%) of exon 2, which results in a constitutive activation of the EGFR pathway and induces a malignant transformation of the cell^6^. SMAD4 is an important tumor suppressor gene in TGF-β pathway, and SMAD4 mutation may lead to an ability to evade apoptosis and deregulation of the cell cycle^10^. This newly established cell line with clear genetic background is an appropriate model for MSS CRC research, including the study of tumorigenesis, metastasis and precision therapy, such as the screening and evaluation of anticancer drugs.

Still more background information is expected. The establishment of tumor cell lines in vitro is actually a process of clonal evolution, why and how chromosomal instability and genetic mutations of the established PUMC-CRC1 cells are higher than that of the original tumor? PUMC-CRC1 cells were derived from one clone, so evolution of mutations generated in different passages during cell culture can be further studied by GWAS. In addition, the transcriptome or proteome profile of the PUMC cells could provide insight to better elucidate CRC.

In conclusion, the original and well characterized human CRC cell line, PUMC-CRC1, was derived from a 47-year-old Chinese female patient diagnosed with colon adenocarcinoma. It is MSS, with KRAS activation, an APC mutation and SMAD4 mutation, and can be used as a model for in vitro and in vivo CRC research of mechanisms of tumorigenesis, metastasis and individualized treatment.

## Methods

### Specimen collection

The clinical specimen was obtained from a 47-year-old Chinese female patient diagnosed with right colon cancer, who underwent right hemicolectomy and didn’t receive any chemotherapy or radiotherapy before surgery. Fresh tumor specimens were obtained from the edge of whole tumor tissues to minimize the necrotic parts during the excision surgery and transported to our laboratory in D-Hank’s buffer supplemented with 2X penicillin–streptomycin (PS, #15140122, Grand Island, NY, USA) for culture, and meanwhile small pieces of tumor tissues and normal tissues were preserved at -80℃ and used for DNA extraction. Other resected tissues were transported to the Department of Pathology for frozen section/paraffin embedding to confirm the diagnosis. The original tumor was an ulcerative tumor, located in the cecum, 4 cm × 4.5 cm in size, solid and invading the serosa with pericolonic lymph-node metastasis. The tumor was histopathologically classified as a moderately to poorly differentiated adenocarcinoma of the colon by pathologists. After surgery, the patient didn’t receive any chemotherapy or radiotherapy, and there was no recurrence after two years follow-up.

### Culture of PUMC-CRC1 cells

All reagents used for cell culture were purchased from Life Technologies Corporation (Grand Island, NY, USA) unless stated otherwise. The tumor specimen was rinsed three times with sterile phosphate-buffered saline (PBS) containing 2X PS, minced into small pieces of 1–3 mm^3^, and enzymatically digested with collagenase (type II 1 mg/ml and type IV 1 mg/ml) (Sigma-Aldrich, St. Louis, MO, USA) at 37 °C for 30 min. Then the separated cells were centrifuged, resuspended in growth medium (DMEM/F12 (1:1) medium containing 10% fetal bovine serum (FBS), 1X insulin-transferrin-selenium (ITS, #41400045) and 1X PS), transferred into a 25-cm^2^ flask and cultured at 37 °C in a humidified incubator containing 5% carbon dioxide. When the primary cultured cells reached 70–80% confluency, G418 (100 μg/ml) (#11811023) was used for 2 weeks, which allowed for the elimination of cancer-associated fibroblasts and enriched tumor cells at an early stage of culture. When the cultured cells reached 80%-90% confluency (approximately 2–3 × 10^6^ cells in a T25 flask), they were subcultured at 1:3–1:6 every 5–7 days, sampled at intervals and stored in liquid nitrogen. During the establishment process, samples were repeatedly collected for cell species identification, STR analysis and Mycoplasma detection, to ensure that there was no contamination with other cells or foreign microorganisms. After 20 passages, it was considered a continuous cell line and named as PUMC-CRC1, and characteristics of this cell line were examined, such as growth characteristics, cytogenetic features, cancer-related genetic mutations, in vivo tumorigenicity and IHC markers of the xenografts.

### Cell proliferation

A 3 ml suspension of 3 × 10^4^/ml exponential phase cells at passage 22 was seeded in four 6-well plates and cultured in growth medium. The next day, cell number in 3 parallel wells was detected by Scepter Handheld Automated Cell Counter (Millipore Corporation, Billerica, MA USA) once a day for 8 times total to obtain a cell growth curve. The population doubling time was calculated using online algorithm software (http://www.doubling-time.com/compute.php). The formula was as follows:

$$ Doubling~\;Time = \frac{{Duration*log\left( 2 \right)}}{{Log\left( {Final~\;Concentration} \right)~{-}~log\left( {Initial~\;Concentration} \right)}} $$

### Mycoplasma detection

Referring to the Chinese Pharmacopoeia^44^, we used the arginine broth culture and semi-fluid culture methods to detect Mycoplasmas in the cell culture supernatant of PUMC-CRC1 cells at P10, P20 and P34 within 2 h. Meanwhile, all samples were also checked by a PCR-based assay^45,46^.

### Cell species identification by PCR

The genomic DNA of PUMC-CRC1 cells (P3, P21, P26 and the original tumor) was extracted using a PureLink Genomic DNA Mini Kit (K1820-02, Invitrogen, Carlsbad, CA, USA) and assessed using a NanoDrop 2000 (ThermoFisher, Grand Island, NY, USA). DNA was amplified with multiplex species-specific primers by PCR as previously described^47^. Meanwhile, the genomic DNA of RD (a human rhabdomyosarcoma cell line), Hepa 1–6 (a mouse hepatocarcinoma cell line), PC-12 (a rat phaeochromocytoma cell line), CHO (a Chinese Hamster ovary cells), MDBK (a bovine kidney cell line), MDCK (a dog kidney cell line), VERO (an African green monkey kidney cell line) and LLC-PK1 (a pig kidney cell line) were used as templates for the positive control for each species. Then specific bands were examined by agarose gel electrophoresis and checked under ultraviolet illumination.

### STR profiling

Eighteen STR loci (D5S818, D21S11, D7S820, CSF1PO, D2S1338, D3S1358, vWA, D8S1179, D16S539, TPOX, TH01, D19S433, D18S51, FGA, D13S317, Penta E, D6S1043 and D12S391) and the amelogenin sex-determining marker were amplified and analyzed by capillary electrophoresis as reported previously^47^. STR data were analyzed using the DSMZ (German Collection of Microorganisms and Cell Cultures) and BMCR (Biomedical Cell Resource Center) online STR databases (http://www.dsmz.de/fp/cgi-bin/str.html, http://cellresource.cn/str/default.aspx).

### Chromosome analysis

Chromosomes of PUMC-CRC1 cells at passage 34 in the exponential growth phase were prepared using a standard air-drying method after treatment with a final concentration of 0.01 μg/ml colcemid for 2 h. A total of 50 metaphase spreads were counted to determine the modal number and karyotypes were analyzed by the G-banding technique. The metaphase spreads slides were scanned by GSL-120 automatic imaging system and analyzed by the CytoVision Version 7.5 Build 72 software (Leica, Richmond, IL, USA).

### Tumorigenicity in SCID mice

In vivo, the tumorigenicity of the PUMC-CRC1 cells at passage 25 was assessed by the ability to form tumors in SCID mice at subcutaneous flank injection sites. PUMC-CRC1 cells in an exponential growth phase were harvested and resuspended in PBS to prepare a cell suspension of 6 × 10^7^ cells/ml. Female SCID mice (n = 5, age 5–6 weeks, body weight 18–20 g) were subcutaneously injected in a 0.1 ml suspension into the right axilla and tumor size was measured with a micrometer caliper once a week when a tumor mass was palpable. The approximate tumor size was calculated using the equation: tumor volume = ab^2^/2 in cm^3^, where a and b are the longest and the shortest diameters of the tumor, respectively. When the tumor diameter was close to 1.5 cm, the mice were euthanized by cervical dislocation and placed in the supine position to separate the subcutaneous tumors which were fixed in 4% formaldehyde for pathological examination. The tumorigenesis experiment was repeated using male SCID mice (n = 5, age 5–6 weeks, body weight 19–21 g). Both female and male mice were purchased from Beijing HFK Bioscience CO., LTD, Beijing, China. Tumor volumes measured at the same time point after inoculation between males and females were compared by *t* test in Excel. A *P*-value < 0.05 was considered to be significant.

### Pathology of xenografts

Tissues of xenografts were fixed in 4% formaldehyde, paraffin embedded and diagnosed in H&E sections. Tissue sections were stained with characteristic markers of CRC, such as CK20 (SD-33), VILLIN(EP163) (Gene Tech, Shanghai, China), CK7(EP16) and CDX2(EP25) (ZSGB-BIO, Beijing, China) and markers related to prognosis and treatment, such as MLH1(GM002), PMS2(EP51), MSH2(RED2), MSH6(EP49), Ki67(MIB-1), P53(DO-7) (Gene Tech), HER2(VENTANA anti-HER2/neu 4B5, Roche, Basel, Switzerland) and EGFR (EP22, ZSGB-BIO). The specific experimental operations were performed on a Link 48 immunohistochemistry autostainer (Dako, Glostrup, Denmark) following the manufacturer’s instructions. The expression was evaluated by an experienced pathologist.

### Gene mutation screening

Genomic DNA samples of PUMC-CRC1 cells at passage 26 and normal tissues of the same patient were screened for 618 specific gene mutations using NGS technology on an Illumina sequencing platform (AccBio, Beijing, China). A list of the 618 genes was showed in Supplementary Table S1. Among 618 genes, 232 genes were related to molecular targeted therapy, such as BRAF, KRAS, NRAS, EGFR and MET. A total of 439 signaling pathway genes were related to immunotherapy, such as microsatellite instability (MSI) and mismatch repair (MMR). 53 genes were related to chemotherapy, such as ABCB1 and DPY. In addition, 123 cancer related genes were detected, such as BRCA1/2 and APC. Gene mutations detected in PUMC-CRC1 and normal tissues of the same patient were nonsynonymous mutations with frequencies less than 1% in “The 1000 Genome Project”, and benign and possibly benign gene variations were filtered out.

### Ethics statements

The study was approved by the Ethics Committee of Institute of Basic Medical Sciences, CAMS, with the patient's informed consent, and all methods were carried out in accordance with relevant guidelines and regulations. The animal experiment was approved by the Animal Ethics Committee of Institute of Basic Medical Sciences, CAMS, performed in accordance with the recommendations of the China Regulations on the Administration of Laboratory Animals. The study was carried out in compliance with the ARRIVE guidelines. Five mice were housed in a cage under specific pathogen-free conditions and a 12-h light/dark cycle at 23 ± 2 °C and 60 ± 10% humidity, and standard food and water were freely available.

## Supplementary Information

Supplementary Information.

## Data Availability

All data generated or analyzed during this study are included in this published article (and its Supplementary Information files). Complying with regulations on management of human genetic resources of China, the cell line can be acquired from BMCR (http://www.cellresource.cn/) and can only be used for scientific research.
