# Supplementary material for: Establishment and characterization of a new human colon cancer cell line, PUMC-CRC1
Source: Sci Rep. 2021 Jun 23;11:13122. doi: 10.1038/s41598-021-92491-7 (PMC8222262; doi:10.1038/s41598-021-92491-7)
Supplement: Supplementary file 1 — Supplementary Information. [file 41598_2021_92491_MOESM1_ESM.pdf]

Supplementary Information

Title: Establishment and characterization of a new human colon cancer cell line, PUMC-CRC1

Authors: Xiaocui Bian[1], Fang Cao[2], Xiaowan Wang[1], Yuhong Hou[1], Haitao Zhao[3,*] & Yuqin Liu[1,*]

**A**							
ABL1	ABL2	ACVR1	ACVR1B	AGO2	AIP	AKT1	AKT2
AKT3	ALK	ALOX12B	AMER1	ANKRD11	APC	AR	ARAF
ARFRP1	ARID1A	ARID1B	ARID2	ARID5B	ASXL1	ASXL2	ATM
ATR	ATRX	AURKA	AURKB	AXIN1	AXIN2	AXL	
**B**							
B2M	BABAM1	BAP1	BARD1	BAX	BBC3	BCL10	BCL11A
BCL2	BCL2L1	BCL2L11	BCL2L2	BCL6	BCOR	BCORL1	BIRC3
BIRC5	BLCAP	BLK	BLM	BMPR1A	BRAF	BRCA1	BRCA2
BRD3	BRD4	BRIP1	BTG1	BTK	BUB1	BUB1B	BUB3
**C**							
C11orf30	CALR	CARD11	CARM1	CASP8	CBFB	CBL	CCND1
CCND2	CCND3	CCNE1	CD274	CD276	CD74	CD79A	CD79B
CDC25C	CDC42	CDC73	CDH1	CDK1	CDK12	CDK2	CDK4
CDK5	CDK6	CDK7	CDK8	CDK9	CDKN1A	CDKN1B	CDKN1C
CDKN2A	CDKN2B	CDKN2C	CEBPA	CENPA	CHD2	CHD3	CHD4
CHEK1	CHEK2	CIC	CREBBP	CRKL	CRLF2	CSDE1	CSF1R
CSF3R	CTCF	CTLA4	CTNNA1	CTNNB1	CUL3	CXCR4	CYLD
CYSLTR2							
**D**							
DAXX	DCUN1D1	DDB2	DDR2	DICER1	DIRAS3	DIS3	DIS3L2
DNAJB1	DNMT1	DNMT3A	DNMT3B	DOT1L	DROSHA	DUSP4	
**E**							
E2F1	E2F3	EED	EGF	EGFL7	EGFR	EIF1AX	EIF4A2
EIF4E	ELF3	EML4	EP300	EPAS1	EPCAM	EPHA3	EPHA5
EPHA7	EPHB1	ERBB2	ERBB3	ERBB4	ERCC1	ERCC2	ERCC3
ERCC4	ERCC5	ERF	ERG	ERRFI1	ESR1	ETV1	ETV6
EWSR1	EXT1	EXT2	EZH1	EZH2			
**F**							
FAM175A	FAM46C	FAM58A	FANCA	FANCB	FANCC	FANCD2	FANCE
FANCF	FANCG	FANCI	FANCL	FANCM	FAS	FAT1	FAT4
FBXW7	FGF10	FGF14	FGF19	FGF23	FGF3	FGF4	FGF6
FGFR1	FGFR2	FGFR3	FGFR4	FH	FLCN	FLT1	FLT3
FLT4	FOLR3	FOXA1	FOXA2	FOXL2	FOXO1	FOXP1	FRS2

FUBP1	FYN						
**G**							
GABRA6	GATA1	GATA2	GATA3	GATA4	GATA6	GID4	GLI1
GLI2	GNA11	GNA13	GNAQ	GNAS	GOPC	GPC3	GPR124
GPS2	GRB2	GREM1	GRIN2A	GRM3	GSK3B	GSTA1	
**H**							
H3F3A	H3F3B	H3F3C	HDAC1	HDAC2	HDAC3	HDAC4	HDAC6
HDAC8	HGF	HIF1A	HIST1H1C	HIST1H2BD	HIST1H3A	HIST1H3B	HIST1H3C
HIST1H3D	HIST1H3E	HIST1H3F	HIST1H3G	HIST1H3H	HIST1H3I	HIST1H3J	HIST2H3C
HIST2H3D	HIST3H3	HLA-A	HLA-B	HNF1A	HOXB13	HRAS	HSD3B1
HSP90AA1							
**I**							
ICOSLG	ID3	IDH1	IDH2	IFNGR1	IGF1	IGF1R	IGF2
IGF2R	IKBKE	IKZF1	IL10	IL7R	IL8	INHA	INHBA
INPP4A	INPP4B	INPPL1	INSR	IRF2	IRF4	IRS1	IRS2
**J**							
JAK1	JAK2	JAK3	JUN				
**K**							
KAT6A	KDM5A	KDM5C	KDM6A	KDR	KEAP1	KEL	KIT
KLF4	KLHL6	KMT2A	KMT2B	KMT2C	KMT2D	KNSTRN	KRAS
**L**							
LATS1	LATS2	LMO1	LRP1B	LRRK2	LYN	LZTR1	
**M**							
MAGI2	MALT1	MAP2K	MAP2K2	MAP2K4	MAP3K1	MAP3K13	MAP3K14
MAP4K1	MAPK1	MAPK3	MAPKAP1	MAX	MCL1	MDC1	MDM2
MDM4	MED12	MEF2B	MEN1	MET	MGA	MITF	MLH1
MPL	MRE11A	MSH2	MSH3	MSH6	MSI1	MSI2	MST1
MST1R	MTOR	MUTYH	MYC	MYCL(MYCL1)	MYCN	MYD88	MYO1B
MYOD1							
**N**							
NAT1	NAT2	NBN	NCOA3	NCOR1	NEGR1	NF1	NF2
NFE2L2	NFKBIA	NKX2-1	NKX3-1	NOTCH1	NOTCH2	NOTCH3	NOTCH4
NPM1	NRAS	NRG1	NSD1	NTHL1	NTRK1	NTRK2	NTRK3
NUF2	NUP93						
**O**							
OPRM1							
**P**							
PAK1	PAK3	PAK7	PALB2	PARK2	PARP1	PARP2	PARP3
PAX5	PBRM1	PDCD1	PDCD1LG2	PDGFRA	PDGFRB	PDK1	PDPK1
PEG3	PGR	PHOX2B	PIK3C2B	PIK3C2G	PIK3C3	PIK3CA	PIK3CB
PIK3CD	PIK3CG	PIK3R1	PIK3R2	PIK3R3	PIM1	PLCG2	PLK1
PLK2	PMAIP1	PMS1	PMS2	PNRC1	POLD1	POLE	PPARG
PPM1D	PPP2R1A	PPP4R2	PPP6C	PRDM1	PRDM14	PREX2	PRKAR1A

PRKCE	PRKCG	PRKCI	PRKD1	PRKDC	PRRT2	PRSS8	PTCH1
PTCH2	PTEN	PTK2	PTP4A1	PTPN11	PTPRD	PTPRS	PTPRT
**Q**							
QKI							
**R**							
RAB35	RAC1	RAC2	RAD21	RAD50	RAD51	RAD51B	RAD51C
RAD51D	RAD52	RAD54L	RAF1	RANBP2	RARA	RARB	RASA1
RASSF1	RASSF8	RB1	RBM10	RECQL	RECQL4	REL	RET
RFWD2	RHBDF2	RHEB	RHOA	RICTOR	RIT1	RNF43	ROCK1
ROS1	RPS6KA1	RPS6KA4	RPS6KB1	RPS6KB2	RPTOR	RRAGC	RRAS
RRAS2	RRM1	RTEL1	RUNX1	RUNX1T1	RUNX2	RXRA	RYBP
**S**							
SDHA	SDHAF2	SDHB	SDHC	SDHD	SESN1	SESN2	SESN3
SETD2	SETD8	SF3B1	SH2B3	SH2D1A	SHH	SHOC2	SHQ1
SLC16A7	SLC19A1	SLIT2	SLX4	SMAD2	SMAD3	SMAD4	SMARCA1
SMARCA4	SMARCB1	SMARCD1	SMO	SMYD3	SNAI1	SNAI2	SNCAIP
SOCS1	SOS1	SOX10	SOX17	SOX2	SOX9	SPEN	SPINK1
SPOP	SPRED1	SPTA1	SRC	SRSF2	STAG2	STAT3	STAT4
STAT5A	STAT5B	STK11	STK19	STK40	SUFU	SUZ12	SYK
**T**							
TAF1	TAP1	TAP2	TBX3	TCEB1	TCF3	TCF7L2	TEK
TERT	TET1	TET2	TGFBR1	TGFBR2	TMEM127	TMPRSS2	TNF
TNFAIP3	TNFRSF14	TNFSF11	TOP1	TOP2A	TP53	TP53BP1	TP63
TPX2	TRAF2	TRAF7	TSC1	TSC2	TSHR	TUBB	TWIST1
TYMS							
**U**							
U2AF1	UPF1						
**V**							
VEGFA	VEGFB	VHL	VTCN1				
**W**							
WEE1	WHSC1	WHSC1L1	WISP3	WNT1	WNT5A	WNT6	WRN
WT1	WWTR1						
**X**							
XIAP	XPA	XPC	XPO1	XRCC2			
**Y**							
YAP1	YES1						
**Z**							
ZBTB2	ZFHX3	ZNF217	ZNF703				

Supplementary Table S1. List of 618 genes screened for mutations by NGS technology in Illumina sequencing platform.

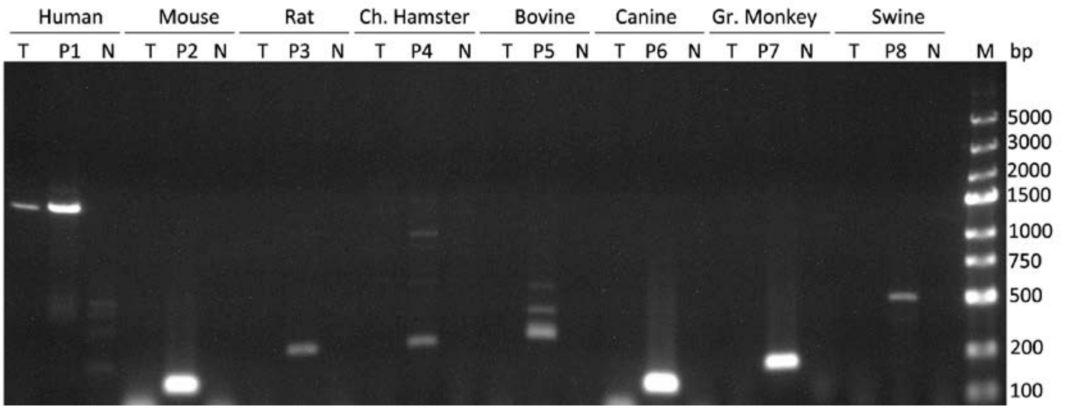

Supplementary Figure S1. PUMC-CRC1 was a human cell line confirmed by a PCR-based method. T, test sample (PUMC-CRC1); N, deionized water used as negative control; P, cell lines of corresponding species used as positive controls, P1, RD (a human rhabdomyosarcoma cell line); P2, Hepa 1-6 (a mouse hepatocarcinoma cell line); P3, PC-12 (a rat phaeochromocytoma cell line); P4, CHO (a Chinese Hamster ovary cells) ; P5, MDBK (a bovine kidney cell line) ; P6, MDCK (a canine kidney cell line); P7, VERO (an African green monkey kidney cell line); P8, LLC-PK1(a swine kidney cell line); M, DNA marker; bp, base pairs. Full-length gel is presented in Supplementary Figure S2.

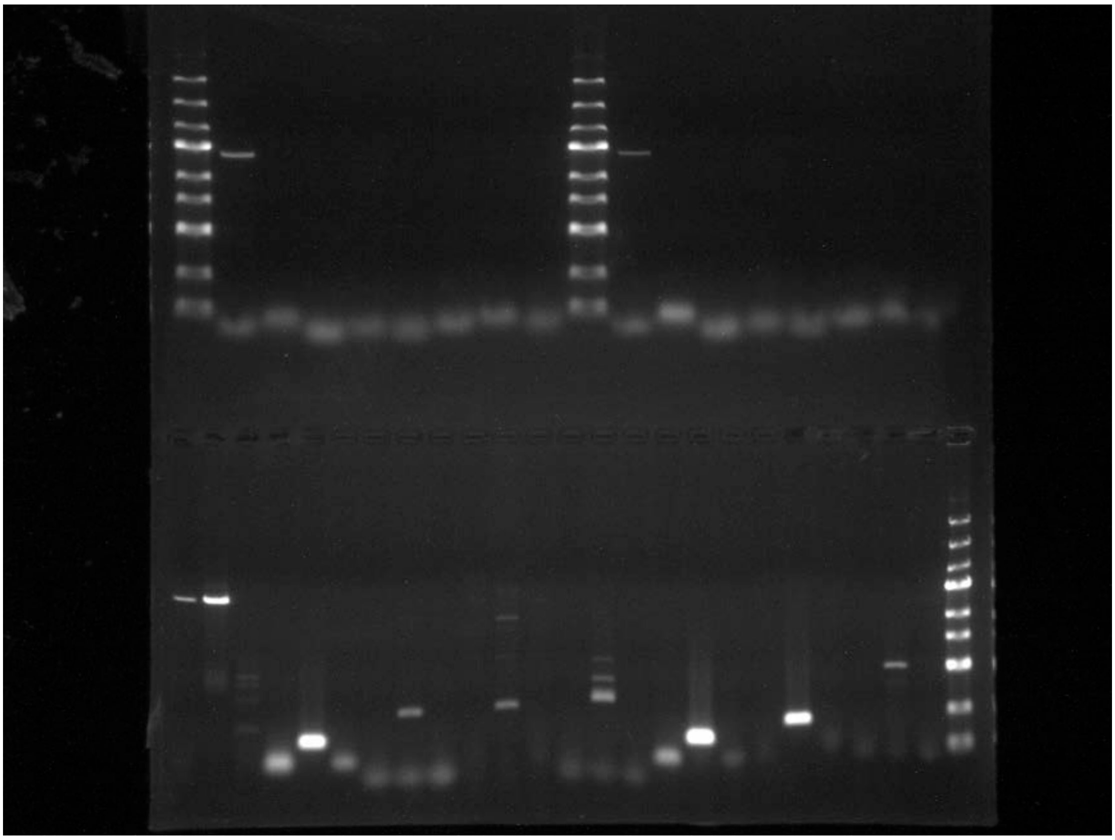

Supplementary Figure S2. Full-length gel picture of Supplementary Figure S1.
